# Supplementary material for: Evolutionary Dynamics of Abundant Stop Codon Readthrough
Source: Mol Biol Evol. 2016 Sep 7;33(12):3108–32. doi: 10.1093/molbev/msw189 (PMC5100048; doi:10.1093/molbev/msw189)
Supplement: Supplementary Data [file supp_33_12_3108__index.html]

Evolutionary Dynamics of Abundant Stop Codon Readthrough — Evolutionary Dynamics of Abundant Stop Codon Readthrough — Supplementary Data 

# Evolutionary Dynamics of Abundant Stop Codon Readthrough

## Supplementary Data

files

- Supplementary Data - zip file
