# Supplementary material for: Evolutionary Dynamics of Abundant Stop Codon Readthrough
Source: Mol Biol Evol. 2016 Sep 7;33(12):3108–32. doi: 10.1093/molbev/msw189 (PMC5100048; doi:10.1093/molbev/msw189)
Supplement: Supplementary Data [file supp_msw189_suppl_data.zip › Supplemental_Text_And_Figures.pdf]

Supplemental Text S1: What we mean by "functional"

To understand what we mean when we refer to "functional" readthrough candidates, it is helpful to review the nature of the signal detected by PhyloCSF. PhyloCSF gets most of its information from substitutions, so regions with very high nucleotide-level conservation have very little signal and tend to get scores near 0, intermediate between the positive scores of most coding regions and negative ones of most noncoding regions. For a region to get a high PhyloCSF score it needs to have many substitutions, and these substitutions need to be ones that make up a higher proportion of the substitutions typical of coding regions than of those typical of noncoding regions, such as synonymous substitutions and substitutions between amino acids with similar biochemical properties. Unlike high amino acid conservation, which could be a side effect of selective constraint on the DNA sequence unrelated to translation, a high PhyloCSF score can only occur when selection has allowed some variation, but has preferred variation that preserves the biochemical properties of a translation product. Furthermore, selective pressure favoring the act of translation rather than its product, say because of some regulatory effect of translation, would have a different signature. Thus, PhyloCSF detects the signature of a fitness advantage of the translation product itself.

Functional Translational Readthrough has been defined as "translational readthrough that leads to functions different from the parent protein" (Schueren and Thoms 2016). While PhyloCSF detects the evolutionary signature of a peptide extension that serves some function, it alone does not show that this function is different from that of the parent protein. However, the fact that leaky stop codon contexts have been conserved for most of our readthrough candidates provides evidence that the extended protein is functionally different from the parent; indeed, if the two were functionally identical then why would there be selective pressure to preserve readthrough and to keep the extension functional?

While these evolutionary signatures provide evidence for Functional Translational Readthrough in evolutionary time, only experimental validation can determine if readthrough of any particular transcript has continued to the present (though SNV data from the *Anopheles gambiae* 1000 genomes project provide evidence for this in the aggregate). Furthermore, while we have evidence that the extended proteins do have functions distinct from the parent, we do not know what their functions are. For these reasons we have referred to them as readthrough *candidates*.

Supplemental Text S2: Estimate for number of orthologous readthrough pairs due to convergent evolution

We estimated that the number of pairs of orthologous readthrough stop codons descended from a non-readthrough ancestral stop codon that have become readthrough independently in the two clades through convergent evolution as roughly the product of the number, N, of non-readthrough ancestral stop codons that have descendent stop codons in the two clades for which we can detect orthology, times the probability, P, that in both clades a non-readthrough ancestral stop codon will have developed readthrough detectable by our process.

We estimate N to be around 7188 by taking the number of stop codons in *A. gambiae* in genes that have a Diptera-level OrthoDB ortholog in *D. melanogaster*, 8562, times the fraction of *A. gambiae* readthrough stop codons having an orthologous gene but not found by orthology, that also have an end-orthologous transcript in *D. melanogaster*, 0.853, minus the number of orthologous readthrough pairs, 115, an estimate for the number to be excluded due to being readthrough in the ancestor. This somewhat overestimates N, because it estimates the number of pairs that are end-orthologous rather than the number satisfying the stricter condition of being stop-orthologous, and because when we subtract orthologous readthrough pairs we ignore the fact that some of our ambiguous readthrough could actually be readthrough. Although it subtracts an estimate for the number of ancestral readthrough stop codons using orthologous readthrough pairs, which treats convergent evolution cases as ancestral, the result is little changed if we do not make this subtraction.

To detect readthrough in both species, we need to detect it in one or the other species without using orthology, but can then use orthology to detect it in the other. We can estimate the probability that a stop codon that was not readthrough in the ancestor has developed readthrough in one species, and that we can detect this readthrough without orthology, by counting the number of non-ancient readthrough candidates in that species (all of which were found without using orthology) and dividing by the total number of stop codons in that species minus the number of ancient readthrough candidates. We can estimate the probability that the orthologous stop codon is readthrough in the other species and that we will be able to detect it, possibly using orthology, using our estimate for the number of readthrough stop codons in each species for which the PhyloCSF-Ψ_{Emp} score of the second ORF is positive, since that is roughly the criteria we used to determine if a stop codon is readthrough once we know it is stop orthologous to a readthrough stop codon in the other species, minus the number of ancient readthrough regions having positive PhyloCSF-Ψ_{Emp}, divided by the total number of stop codons in that species minus the number of ancient readthrough candidates. Carrying out these estimates we find that the probability that a non-readthrough ancestral stop codon will have become readthrough in both clades is approximately 0.0008.

However, while this calculation accounts for the fact that *detection* of readthrough in one species helps us detect readthrough in the other, it does not take into account that some genes, such as longer ones, could have a greater tendency to *become* readthrough, in both species, than others. Accounting for the distributions of first ORF lengths of readthrough candidates and of all other genes increases the probability that a non-readthrough ancestral stop codon will have developed readthrough in both clades by about 30%, which gives us an estimate for P of 0.00104.

Combining these estimates of *N* and *P*, we estimate that the number of pairs of orthologous readthrough stop codons that have become readthrough independently from a non-readthrough ancestral stop codon through convergent evolution in the two clades is around 7188 * .00104 = 7.48.

A caveat is that there could be unknown confounders other than first ORF length that make an ancestral gene more likely to develop readthrough, though such confounders would have be to considerably more influential than first ORF length to change the result much in comparison to the total number of ancient readthrough regions.

Supplemental Text S3: Z curve test provides an underestimate for the number of readthrough regions

There are several reasons that the results of our Z curve test is likely to be an underestimate for the actual number of functional readthrough regions.

First, the test only counts readthrough regions at least 10 codons long and that have Z curve score greater than 0. As discussed in the main text, using our PhyloCSF test we found evidence that more than half of functional readthrough regions are less than 10 codons long. Furthermore, only about half of the readthrough regions of our readthrough candidates have Z curve score > 0 (Supplemental Figure S5C,D). Since the readthrough regions of our readthrough candidates are the ones having the highest coding potential as measured by PhyloCSF, we would expect them also to have higher Z curve scores than most of the other readthrough regions, so probably fewer than half of functional readthrough regions have Z curve score > 0. Combining these results, we find that the estimate provided by our test is probably less than 25% of the actual number of annotated stop codons that have functional readthrough.

Second, our test only looks at annotated stop codons, which could be considerably fewer than the number of actual stop codons in species having incomplete annotations.

Third, there are several factors that could inflate the counts of second ORFs having positive Z curve scores in frames 1 and 2 but not frame 0, which would decrease any frame-0 excess and thus our estimate. When an annotated stop codon lies within a coding region in another frame, the second ORF of that stop codon in the other frame is likely to have a high Z curve score, which would inflate the count in frame 1 or 2; we exclude any stop codons that are within *annotated* coding regions in another frame, but stop codons within *unannotated* coding regions in other frames would bias our readthrough estimate downward. Also, while our estimate corrects for recent nonsense substitutions and sequencing errors that read a sense codon as a stop codon, which would inflate the count in frame 0, we do not correct for recent indels and sequencing indels, which would inflate the counts in frames 1 and 2 and bias our readthrough estimate downward.

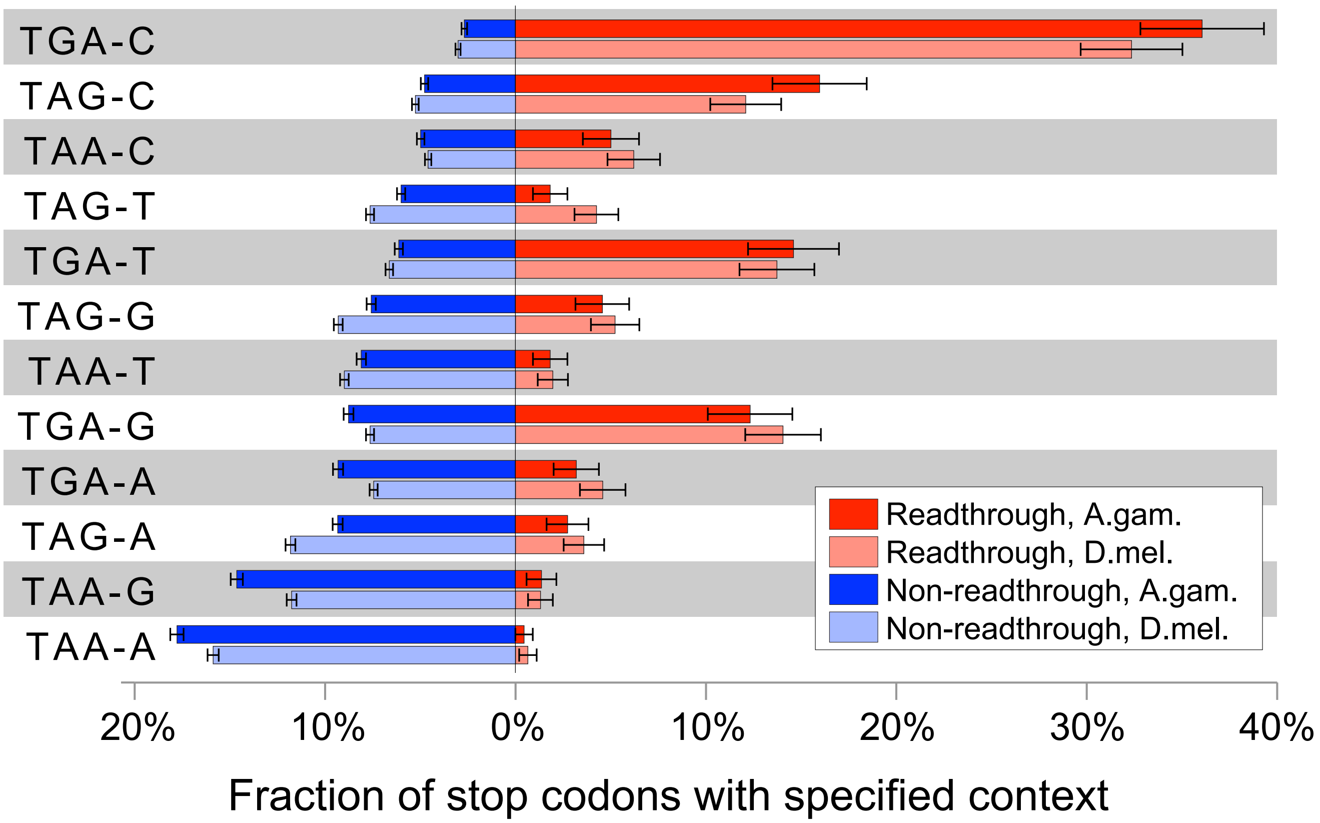

Supplemental Figure S1. Stop codon contexts in *A. gambiae* and *D. melanogaster*. Usage of stop codon context (stop codon and subsequent base) sorted in order of decreasing frequency among the 12,058 non-readthrough, non-mitochondrial, annotated *A. gambiae* stop codons (dark blue), with less frequent stop codons (top, e.g., TGA-C) experimentally associated with translational leakage in other species, and most frequent (bottom, e.g., TAA-A) associated with efficient termination. Error bars show standard error of mean. Context frequencies for *A. gambiae* readthrough candidates (red) are almost the opposite of those of non-readthrough transcripts, suggesting a preference for leaky context, with 36% using TGA-C and almost none using TAA-A. Frequencies shown are for our “unbiased by stop codon” subset of readthrough candidates and exclude double-stop readthrough candidates. The frequencies for both readthrough and non-readthrough stop codons are similar to those for *D. melanogaster* (pink and light blue, respectively).

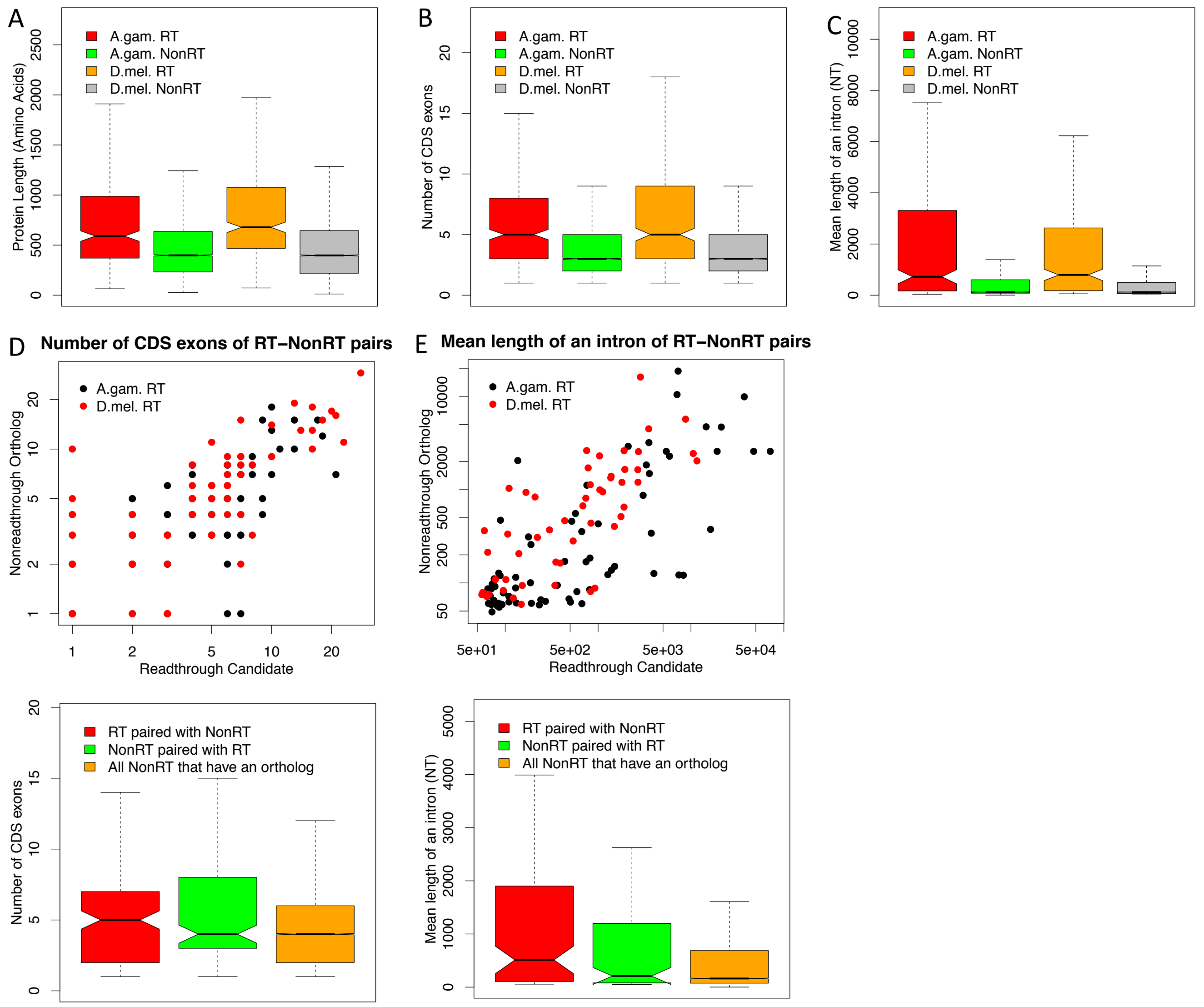

Supplemental Figure S2. Readthrough genes are longer than non-readthrough. (A-C) Box plots for lengths of unextended proteins (A), number of exons in the coding portion of the transcript (B), and average length of an intron in each transcript having at least one intron (C), for *A. gambiae* readthrough candidates (red), *A. Gambia* non-readthrough transcripts (green), *D. melanogaster* readthrough candidates (orange), and *D. melanogaster* non-readthrough transcripts (gray). By all three measures, readthrough candidates are significantly longer than non-readthrough transcripts, in both species. (D,E) Upper panels are scatter plots showing number of coding exons (D) and average intron length (E) for each *A. gambiae* readthrough candidate orthologous to a non-readthrough *D. melanogaster* transcript versus the corresponding value for its non-readthrough ortholog (black dots) and similarly for *D. melanogaster* readthrough candidates orthologous to non-readthrough *A. gambiae* transcripts (red dots). Lower panels are box plots showing number of coding exons (D) and average intron length (E) of those readthrough candidates in either species that are orthologous to a non-readthrough transcript in the other (red), the corresponding lengths of the paired non-readthrough transcripts (green), and the lengths of all non-readthrough transcripts in genes that have orthologs in the other species (orange). Unlike the similar comparison for protein lengths shown in Figure 3E, there is no clear relationship among the number of exons or average intron length of readthrough candidates, their non-readthrough orthologs, and other non-readthrough transcripts.

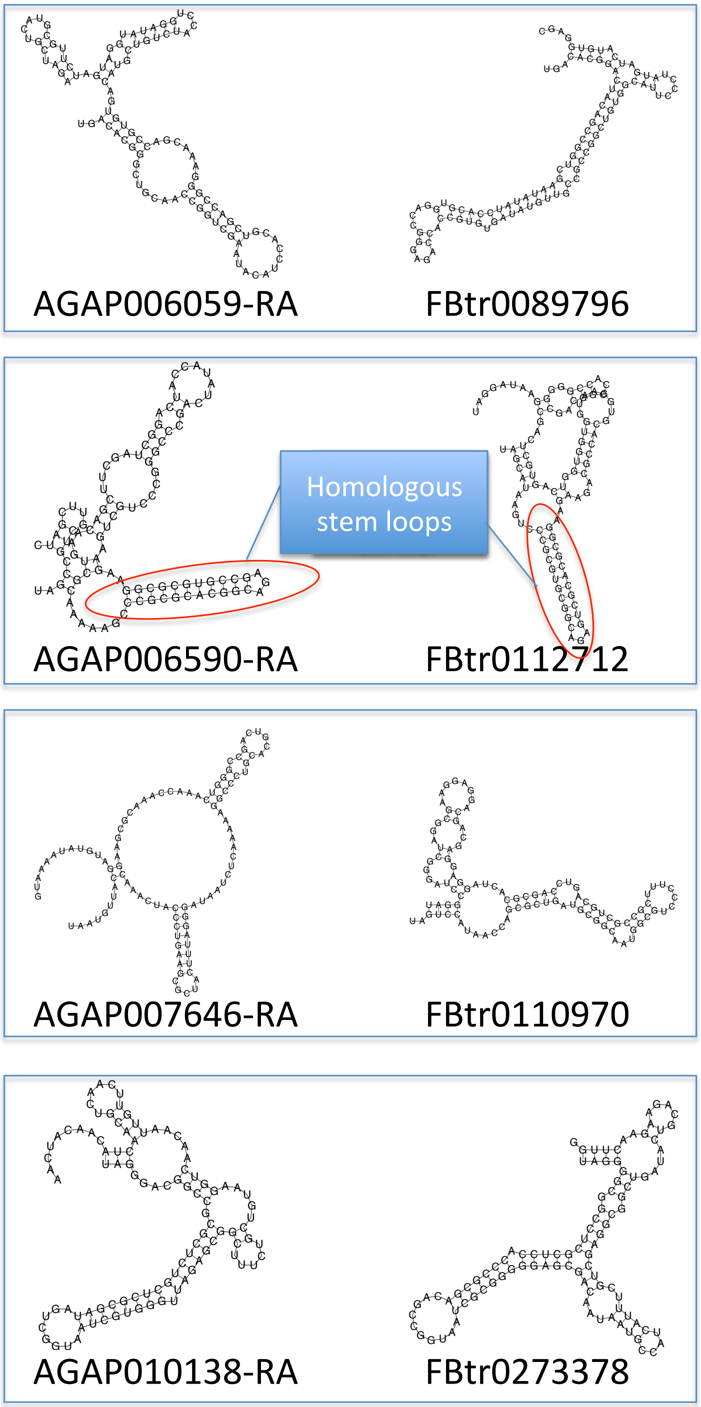

Supplemental Figure S3. RNA structures for readthrough-readthrough pairs. Conserved, stable, RNA structures predicted by RNAz in the 100-nt regions downstream from (and including) candidate readthrough stop codons for the four stop-orthologous pairs of readthrough candidates in *A. gambiae* (left) and *D. melanogaster* (right) for which a structure was predicted in both. There is clear homology between stem loops near the 5' ends of the second pair of structures (red ovals). Other than that, we see no obvious similarity between the predicted structures in the two species, offering the possibility that it is the presence of a stable structure that is functional rather than particular features of that structure.

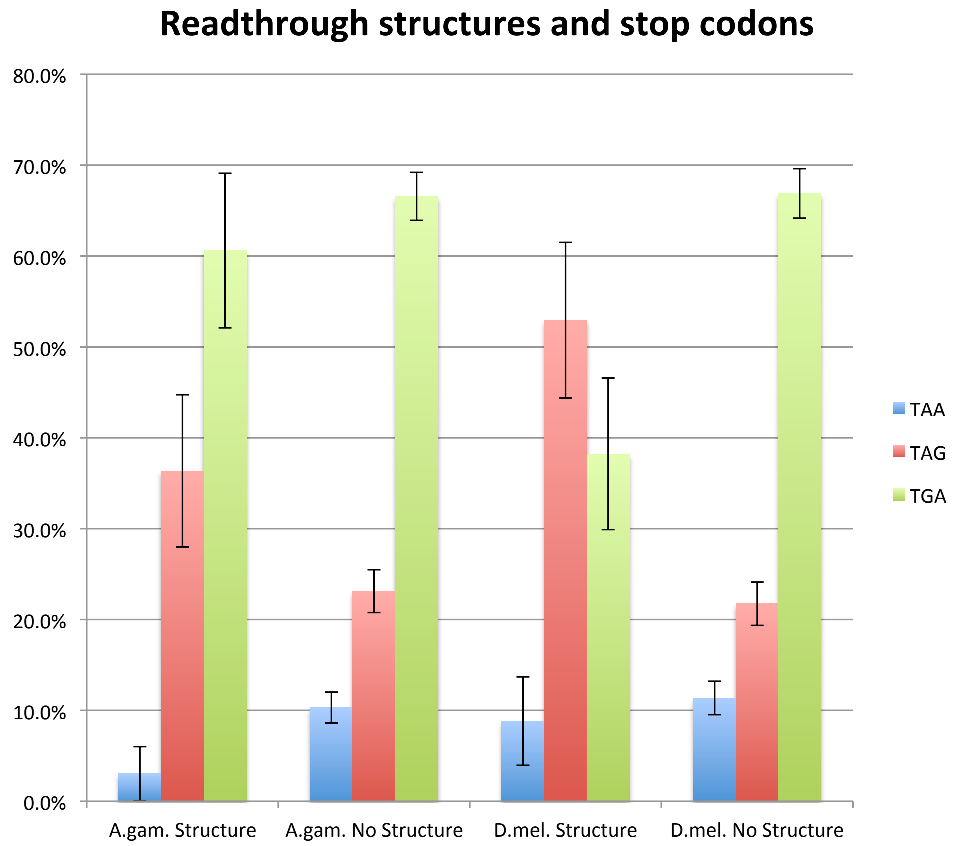

Supplemental Figure S4. Stop codon usage in candidates having predicted structures. Frequencies of usage of TAA (blue), TAG (red), and TGA (green) first stop codons among *A. gambiae* readthrough candidates having predicted structures (first group), *A. gambiae* readthrough candidates lacking predicted structures (second group), *D. melanogaster* readthrough candidates having predicted structures (third group), and *D. melanogaster* readthrough candidates lacking predicted structures (fourth group), with error bars showing standard error of mean. Although most readthrough candidates in both species use TGA, readthrough candidates having structures in *D. melanogaster* show a preference for TAG, prompting speculation that a leaky stop codon context might not be necessary for readthrough in the presence of an RNA structure. However, in *A. gambiae* there is only a small and not-statistically significant depletion for TGA stop codons among readthrough candidates having predicted structures.

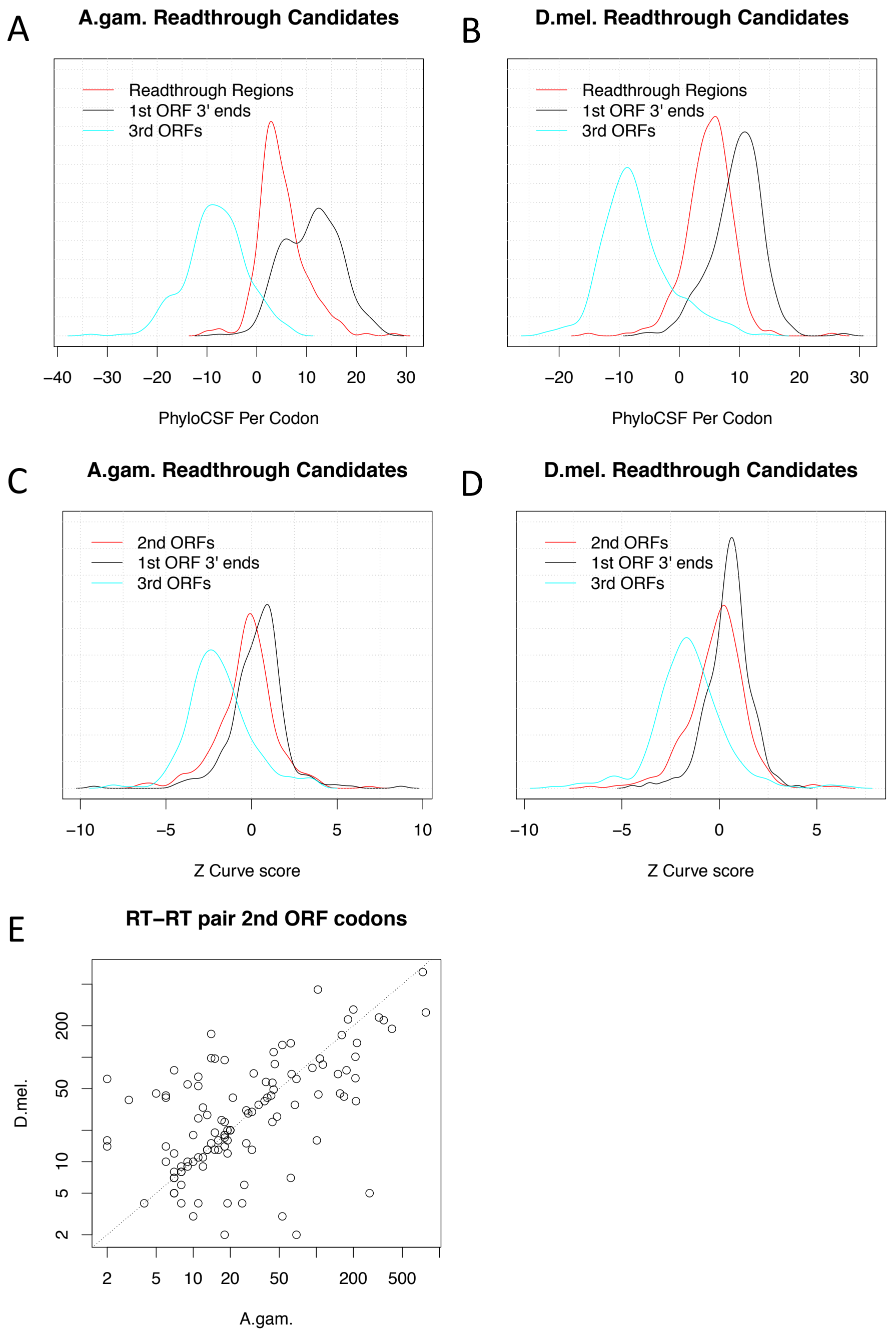

Supplemental Figure S5. Readthrough regions under less constraint than other coding regions. (A-D) Distribution of coding potential as measured by PhyloCSF score per codon (A and B) or Z curve score (C and D) of readthrough regions (red), same-sized coding regions at the 3' end of the first ORF of readthrough candidates (black), and noncoding third ORFs of readthrough candidates (cyan), for *A. gambiae* (A and C) and *D. melanogaster* (B and D). Double readthrough candidates and readthrough candidates whose third ORF is 0-length or contains degenerate bases have been excluded. In all four cases, coding potential of readthrough regions tends to be intermediate between that of noncoding regions and other coding regions, suggesting that they have been under some purifying selection at the amino acid level within each clade, but less so than other coding regions. (E) Log-scale scatter plot showing the lengths in codons of the readthrough regions in *A. gambiae* and *D. melanogaster* for each pair of stop-orthologous readthrough candidates. Although for many pairs the lengths are similar in the two species (circles along diagonal line), there are also many pairs for which the lengths are quite different, suggesting that in many cases readthrough regions have remained functional despite large changes in length.

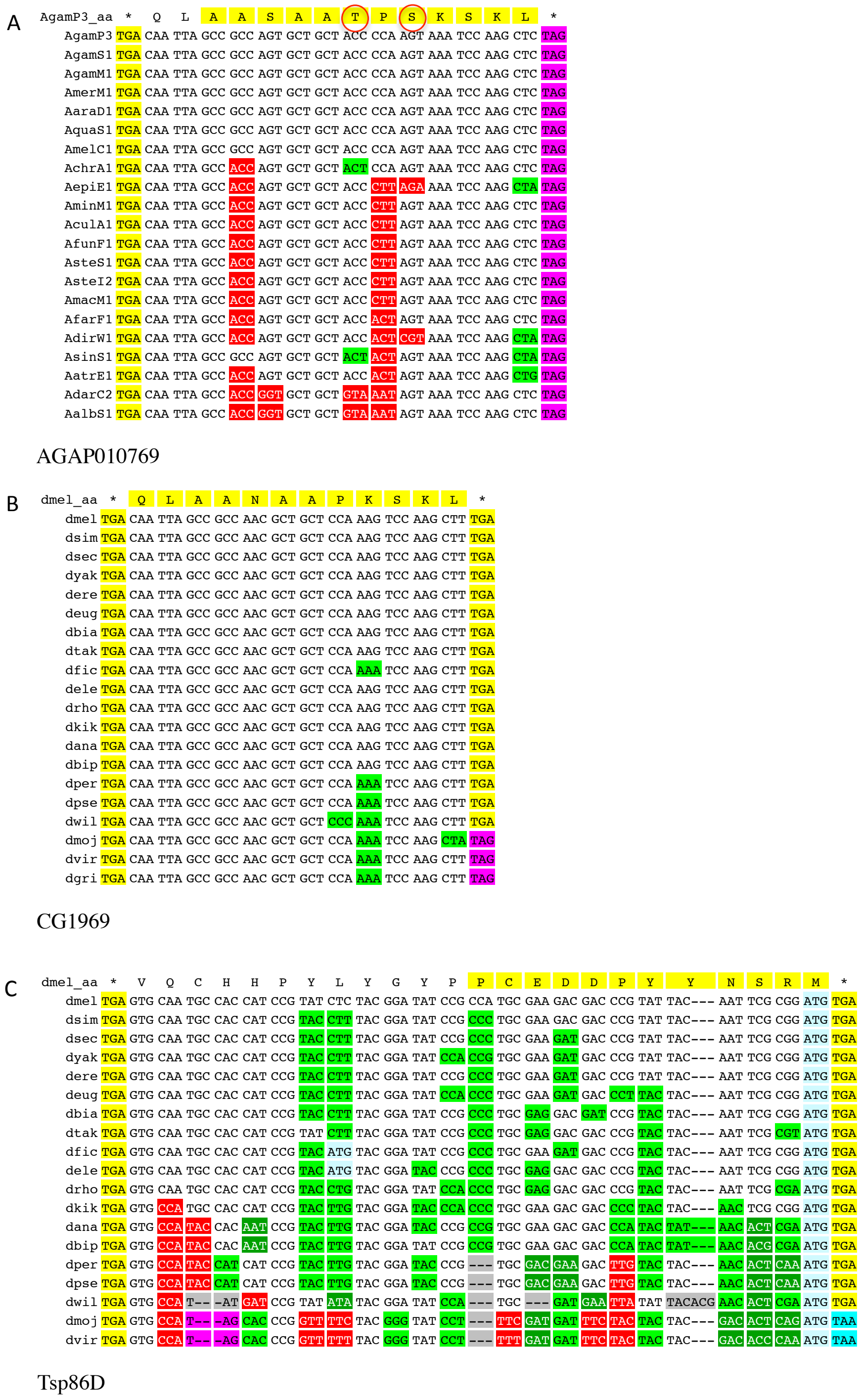

Supplemental Figure S6. Peroxisomal targeting signals. Alignments of readthrough regions of *A. gambiae* gene AGAP010769 among 21 *Anopheles* genomes (A), its *D. melanogaster* ortholog, CG1969, among 20 *Drosophila* genomes (B), and *D. melanogaster* gene *Tsp86D* (C). A predicted peroxisomal targeting signal in the final 12 amino acids of the extension of AGAP010769 (yellow highlighting) has been conserved across all 21 mosquitoes and 20 flies, despite the presence of several radical amino acid substitutions within the *Anopheles* lineage (red highlighting) and two amino acid insertions or deletions between the two clades (red circles). The predicted peroxisomal targeting signal in *Tsp86D* is conserved as far as *D. kikkawai* but not in *D. ananassae* or beyond.

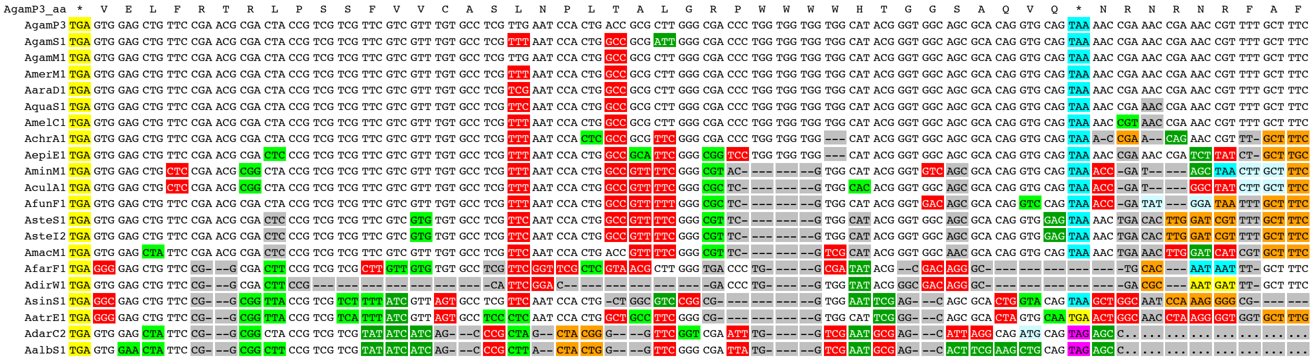

Supplemental Figure S7. Readthrough candidates identified using other features of coding regions. Alignment including readthrough region of *A. gambiae* readthrough candidate AGAP008312-RA. Although the PhyloCSF+Stop score of this readthrough region, 10.9, is below our threshold of 17.0, it was included among our list of readthrough candidates because it exhibits other features characteristic of coding regions that are not accounted for by PhyloCSF+Stop. In particular, the reading frame is preserved in all species despite the presence of many indels (gray) but degrades soon after the second stop codon (orange) and there are several synonymous substitutions in the second stop codon. (All insertions relative to *A. gambiae* before the second stop codon are frame preserving but are not shown in order to make the image more compact.) We included 40 second ORFs having PhyloCSF+Stop between 5.0 and 17.0 in our list of *A. gambiae* readthrough candidates based on a subjective assessment of these and other features characteristic of protein coding regions.

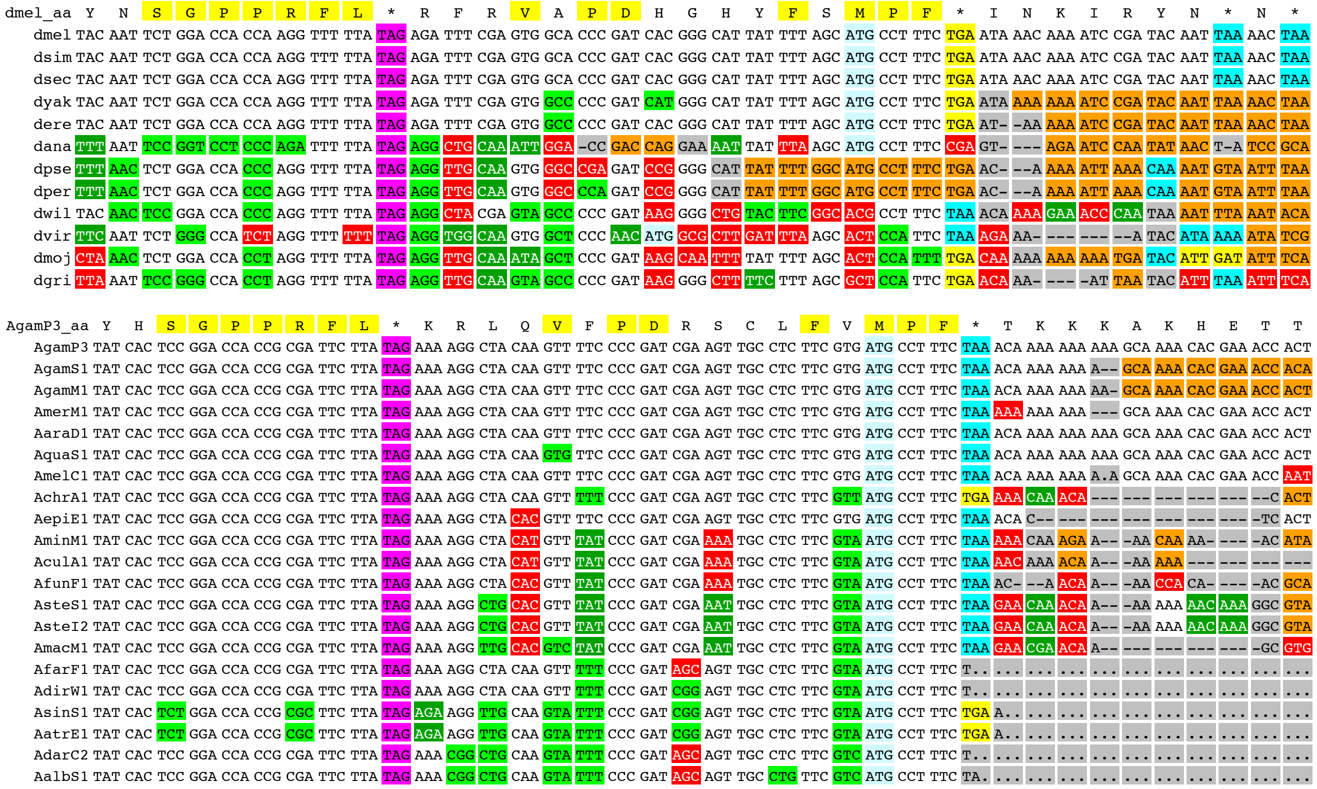

Supplemental Figure S8. Readthrough identified using orthology. Alignment of the readthrough region of *D. melanogaster* readthrough candidate FBtr0340041 (upper panel) identified using orthology to *A. gambiae* readthrough candidate AGAP002951-RA (lower panel), with many matching amino acids both within the readthrough region and before the first stop codon (yellow highlighting). FBtr0340041 had not been previously identified as readthrough, but satisfied our criteria for readthrough given orthology to a readthrough candidate in the other clade. In order to facilitate cross-clade comparisons, we identified 51 *D. melanogaster* and 21 *A. gambiae* readthrough candidates in this way.

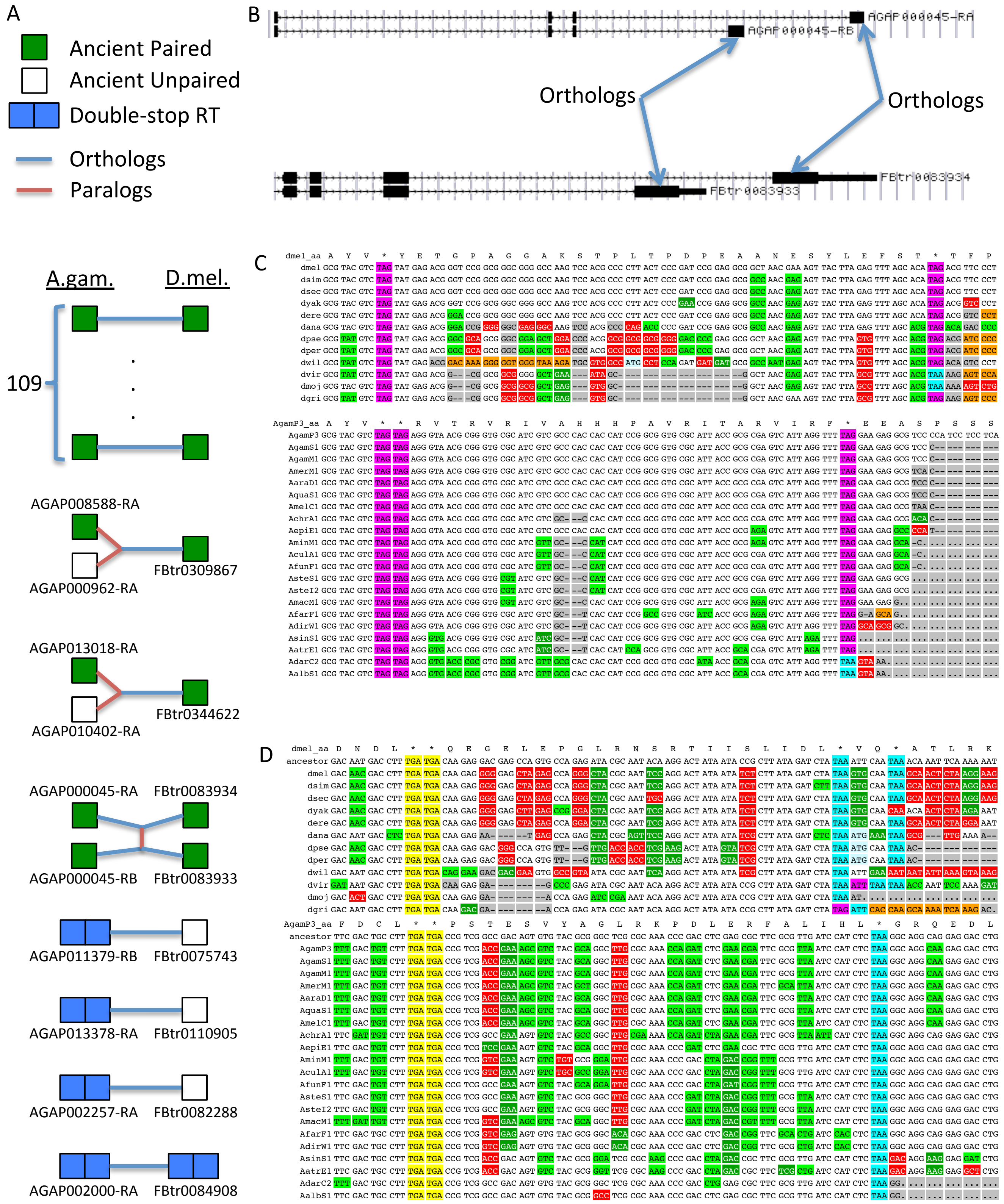
A
Ancient Paired
Ancient Unpaired
Double-stop RT
Orthologs
Paralogs
A.gam.
D.mel.
109
AGAP008588-RA
AGAP000962-RA
FBtr0309867
AGAP013018-RA
AGAP010402-RA
FBtr0344622
AGAP000045-RA
FBtr0083934
AGAP000045-RB
FBtr0083933
AGAP011379-RB
FBtr0075743
AGAP013378-RA
FBtr0110905
AGAP002257-RA
FBtr0082288
AGAP002000-RA
FBtr0084908
B
AGAP000045-RA
AGAP000045-RB
Orthologs
Orthologs
FBtr0083934
FBtr0083933
C
D

Supplemental Figure S9. Ancient readthrough pairing. (A) Pairing of ancient readthrough candidates in *A. gambiae* and *D. melanogaster*. We defined a readthrough candidate to be "ancient" if it is stop-orthologous to a readthrough candidate in the other species at the *Diptera* level and is not a double-stop readthrough candidate. In most cases (109) there is a one-to-one pairing of orthologous ancient readthrough candidates (green boxes). However, in two cases there are two paralogous *A. gambiae* readthrough candidates orthologous to the same *D. melanogaster* candidate, so we excluded one of the paralogs (white boxes) from analyses that required a one-to-one pairing. There is one case of four homologous readthrough candidates, however we were able to determine that these split into two orthologous pairs (panel B). There are three *D. melanogaster* readthrough candidates orthologous to double-stop readthrough candidates in *A. gambiae* (blue rectangles), so these were excluded from analyses that required a one-to-one pairing of non-double-stop readthrough candidates (panel C). Finally, there is one pair of orthologous double-stop readthrough candidates, which were excluded from most of our analyses (panel D). (B) Four-way homologous readthrough candidates. Two *A. gambiae* readthrough candidates and two *D. melanogaster* readthrough candidates, all homologous at the *Diptera* level, displayed using the UCSC genome browser. In each species, the first ORFs of the two readthrough candidate transcripts are four-exon alternative splice variants of a single gene, with the first three exons shared. The downstream fourth exons in the two species are more similar to each other than either is to the alternative exons, and the same is true of the upstream fourth exons, suggesting that these two genes are descended from a gene in the common ancestor having a similar configuration, and that the alternative final exons were formed by an earlier duplication of a final exon containing a readthrough stop codon. (C) Double-stop readthrough orthologous to single-stop readthrough. Alignments including the readthrough regions of *D. melanogaster* readthrough candidate FBtr0075743 and *A. gambiae* double-stop readthrough candidate AGAP011379-RB. It is possible that the second TAG stop codon in the double first stop codon of AGAP011379-RB is related by a single nucleotide substitution to the TAT tyrosine codon immediately 3' of the first stop codon in FBtr0075743. We note that although there is very little cross-clade similarity between the readthrough regions at the amino acid level, within each of the two clades there are several alignment columns immediately after the first stop codon and near the end of the readthrough region that have no synonymous substitutions, suggesting that there might be some overlapping constraint at the nucleotide level in addition to any constraint on the amino acid sequences. (D) Orthologous double-stop readthrough candidates. Alignments including the readthrough regions of orthologous double-stop readthrough candidates FBtr0084908 and AGAP002000-RA. The common double-TGA stop codon suggests that these might be descended from a double-stop readthrough transcript in the common ancestor.

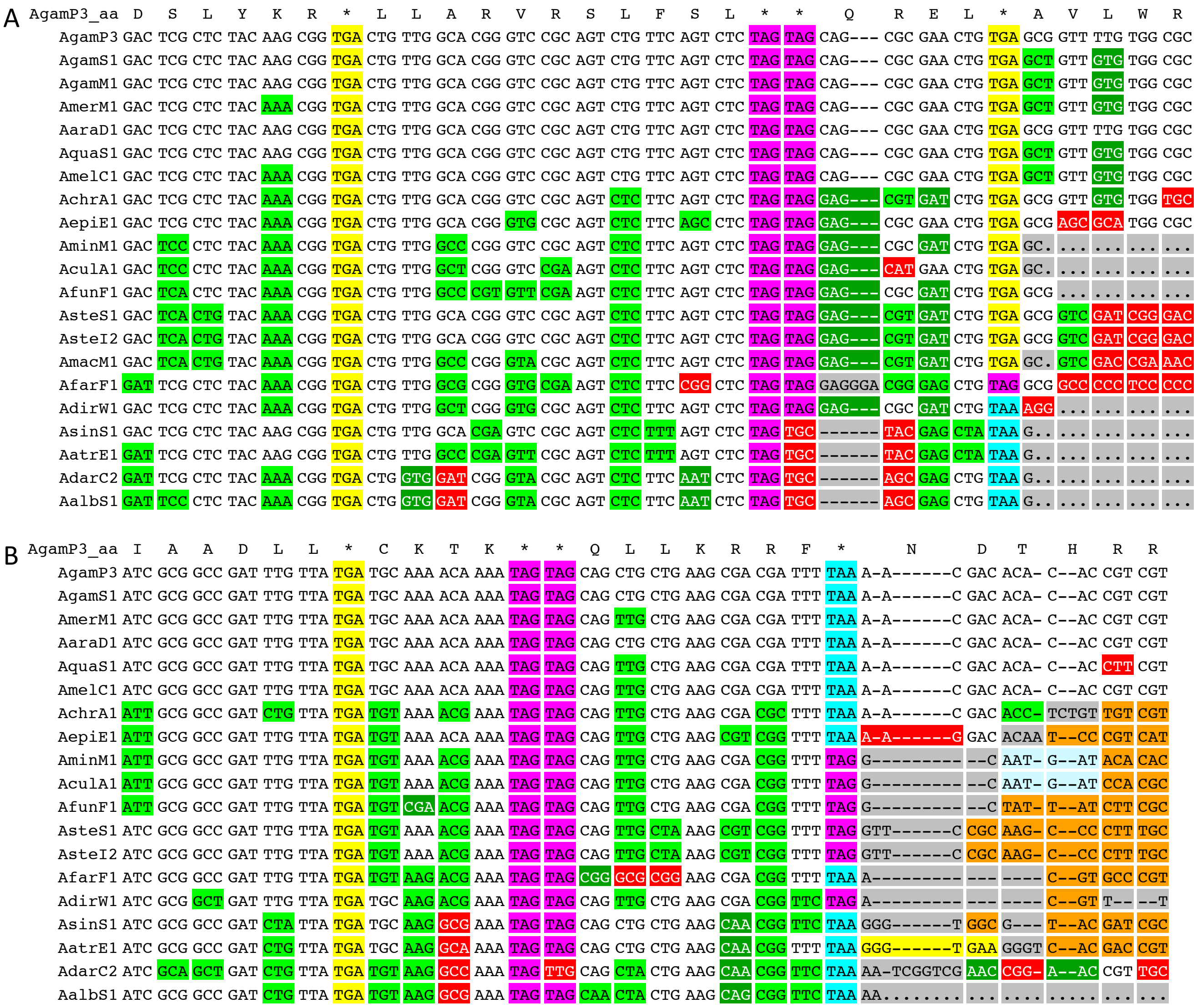

Supplemental Figure S10. Single-double readthrough. Alignments showing triple readthrough for AGAP001806-RA (A) and AGAP012372-RA (B), in which a single readthrough is followed by a double-stop readthrough in some species. In AGAP001806-RA, the second TAG stop codon in the *A. gambiae* double stop codon is aligned to a likely-ancestral TGC Cysteine codon in several species, though the presence of indels makes the history of the particular codon uncertain. In AGAP012372-RA the second TAG stop codon in the double stop codon appears to be ancestral, and has become a TTG Leucine codon in *A. darlingi*.

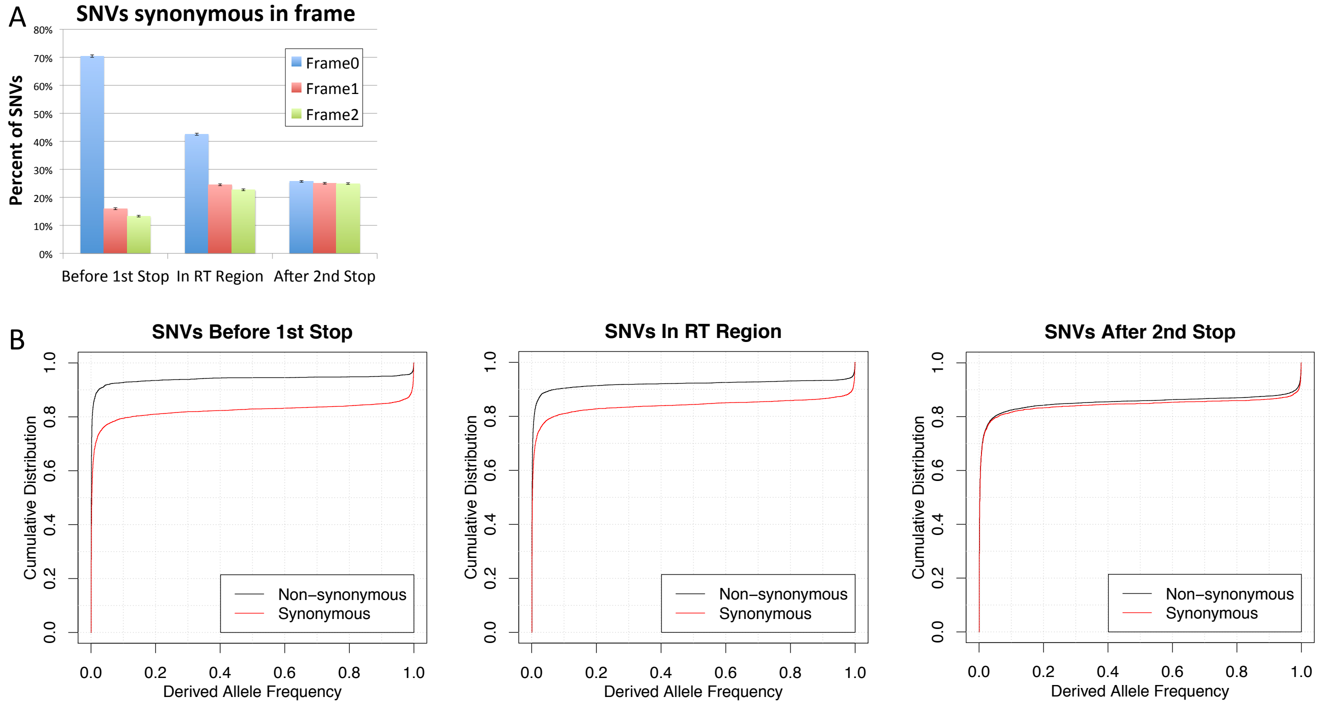

Supplemental Figure S11. Polymorphism evidence supports recent protein-coding selection. (A) Single nucleotide variants (SNVs) in *A. gambiae* show a strong bias toward synonymous codon changes in readthrough regions (middle) and same-sized coding regions 5' of readthrough stop codons (left), but not in same-sized noncoding regions 3' of second stop codons of readthrough candidates (right), providing evidence that readthrough regions are under purifying selection at the amino acid level within the *A. gambiae* population. For each type of region we show the fraction of SNVs that would be synonymous if translated in each of three frames, with frame 0 matching the translated frame of the coding region of the gene. Error bars show the Standard Error of the Mean (SEM). (B) Cumulative distributions of derived allele frequencies for SNVs that would be synonymous (red) or non-synonymous (black) if translated in the frame of the coding region of the gene, for the same three sets of regions. Derived allele frequencies are lower for non-synonymous SNVs than for synonymous ones, in both coding and readthrough regions, indicating that they are under greater purifying selection, whereas in noncoding regions there is no significant difference, providing further evidence that purifying selection at the amino acid level in readthrough regions has continued in the *A. gambiae* population.

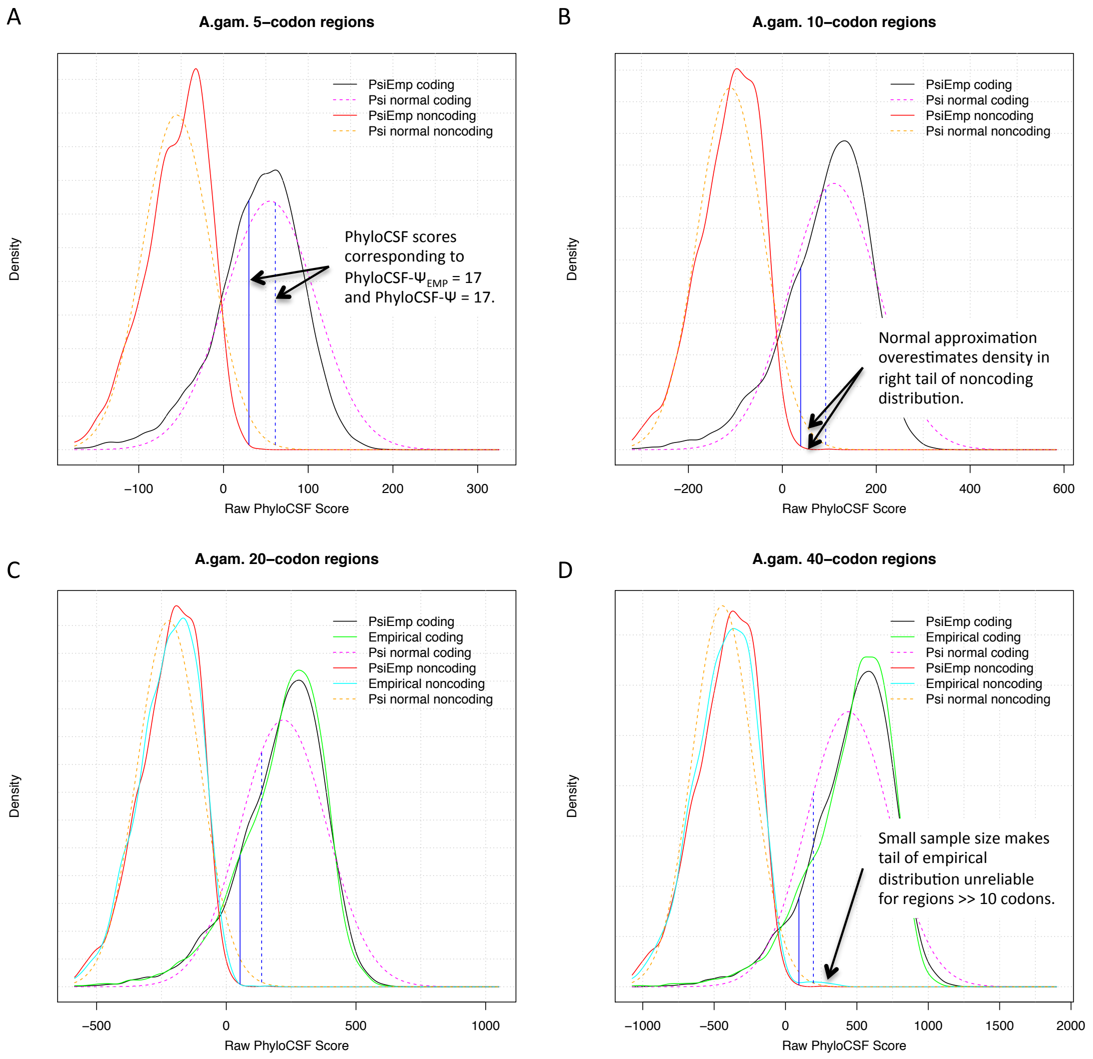
A
A.gam. 5–codon regions
PsiEmp coding
Psi normal coding
PsiEmp noncoding
Psi normal noncoding
PhyloCSF scores corresponding to PhyloCSF-Ψ_EMP = 17 and PhyloCSF-Ψ = 17.
Density
Raw PhyloCSF Score
B
A.gam. 10–codon regions
PsiEmp coding
Psi normal coding
PsiEmp noncoding
Psi normal noncoding
Normal approximation overestimates density in right tail of noncoding distribution.
Density
Raw PhyloCSF Score
C
A.gam. 20–codon regions
PsiEmp coding
Empirical coding
Psi normal coding
PsiEmp noncoding
Empirical noncoding
Psi normal noncoding
Density
Raw PhyloCSF Score
D
A.gam. 40–codon regions
PsiEmp coding
Empirical coding
Psi normal coding
PsiEmp noncoding
Empirical noncoding
Psi normal noncoding
Small sample size makes tail of empirical distribution unreliable for regions >> 10 codons.
Density
Raw PhyloCSF Score

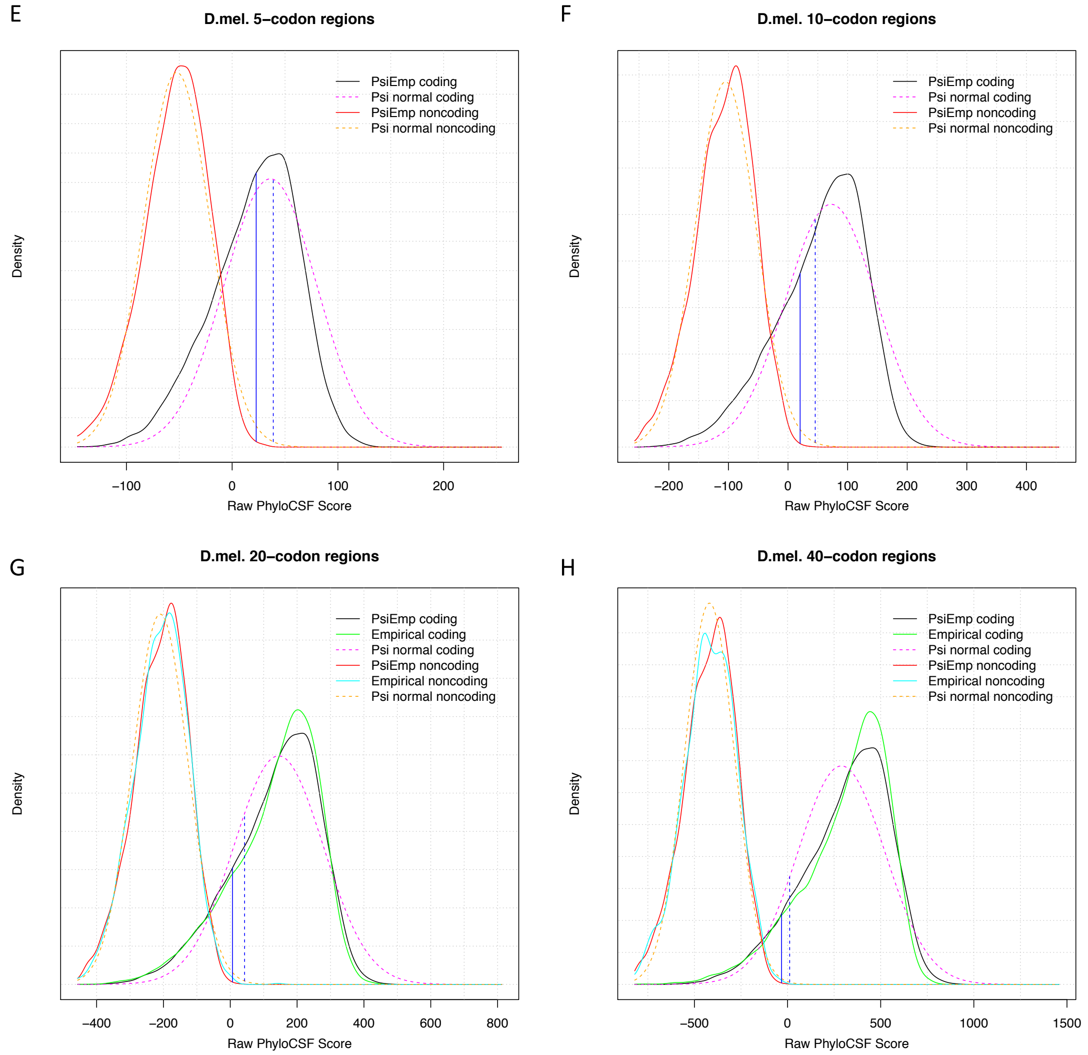

Supplemental Figure S12. Empirical score distributions allow PhyloCSF-Ψ_{Emp} to achieve higher sensitivity than PhyloCSF-Ψ while maintaining high specificity. Coding (black) and noncoding (red) PhyloCSF score distributions used to define PhyloCSF-Ψ_{Emp} for *A. gambiae* (A-D) and *D. melanogaster* (E-H), and normal approximations used to define PhyloCSF-Ψ (magenta and orange dashed lines), for regions of lengths 5, 10, 20, and 40 codons. For lengths less than or equal to 10, the distributions used to define PhyloCSF-Ψ_{Emp} were calculated directly from training regions of that length, whereas for greater lengths PhyloCSF-Ψ_{Emp} was defined by scaling the distributions for regions of length 10. For lengths greater than 10 we show the actual distributions of coding (green) and noncoding (cyan) training regions of those lengths for reference, even though they were not used in calculating PhyloCSF-Ψ_{Emp}. The bump in the right tail of the cyan curve for *A. gambiae* 40-codon regions (D) is presumably a result of sampling error due to the small number of noncoding training regions of that length; such sampling errors are the reason that we scaled the length-10 distribution rather than interpolating densities through scores of actual regions of greater lengths. PhyloCSF scores for regions of each length for which PhyloCSF-Ψ_{Emp} and PhyloCSF-Ψ are equal to our score threshold of 17 (solid and dashed vertical blue lines, respectively) are the scores that are approximately 50 times more likely to occur in coding regions than noncoding regions. Because the normal approximations overestimate the densities in the right tail of the noncoding score distributions, PhyloCSF-Ψ overestimates the PhyloCSF score needed to achieve this high specificity; by using the more accurate empirical densities, PhyloCSF-Ψ_{Emp} allows us to detect coding regions having lower PhyloCSF score, achieving greater sensitivity while maintaining this high specificity.

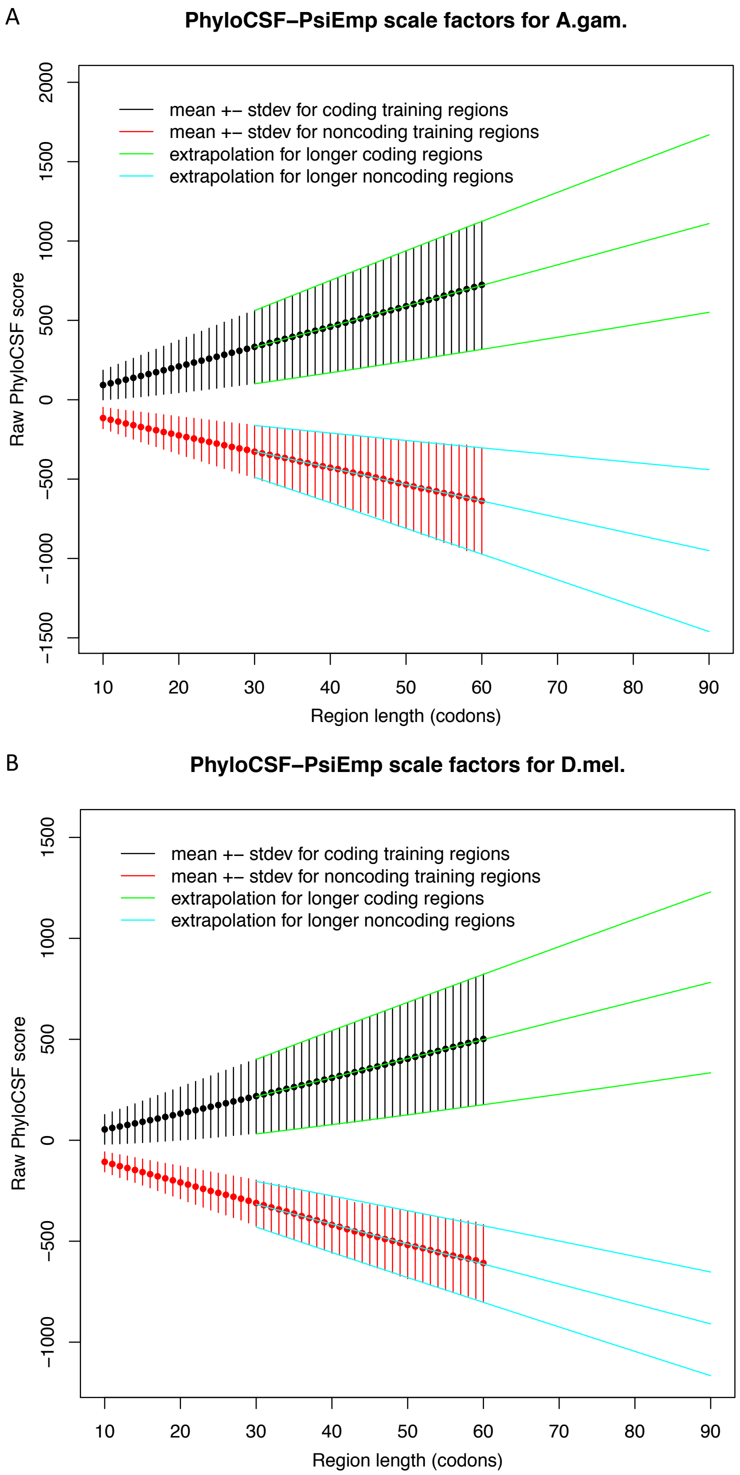

Supplemental Figure S13. Score distribution scale factors used for definition of PhyloCSF-Ψ_{Emp}. Mean PhyloCSF scores of coding (black circles) and noncoding (red circles) training regions of each length used to define PhyloCSF-Ψ_{Emp} for *A. gambiae* (A) and *D. melanogaster* (B) and intervals one standard deviation above and below (black and red vertical lines). For lengths greater than 10 codons, PhyloCSF-Ψ_{Emp} was defined by scaling the distributions for regions of length 10 codons using the means and standard deviations of the scores of training regions of each length up to 60 codons, and an extrapolation using linear regression on the means and the logs of the standard deviations of regions of length from 30 to 60 codons for regions greater than 60 codons (green and cyan curves).
