# Supplementary material for: Evolutionary Dynamics of Abundant Stop Codon Readthrough
Source: Mol Biol Evol. 2016 Sep 7;33(12):3108–32. doi: 10.1093/molbev/msw189 (PMC5100048; doi:10.1093/molbev/msw189)
Supplement: Supplementary Data [file supp_msw189_suppl_data.zip › Supplemental_Table_S1.pdf]

Supplementary Table 1: Genome sources for species in Figure 5

LatinName	CommonName	Version	AnnotationFile	Source	Publication
Drosophila melanogaster	Fruit Fly	5.57	dmel-all-r5.57.gff	flybase.org	(Adams et al. 2000)
Drosophila sechellia	Fruit Fly	1.3	dsec-all-r1.3.gff	flybase.org	(Drosophila 12 Genomes Consortium et al. 2007)
Drosophila simulans	Fruit Fly	2.01	dsim-all-r2.01.gff	flybase.org	(Drosophila 12 Genomes Consortium et al. 2007)
Drosophila erecta	Fruit Fly	1.04	dere-all-r1.04.gff	flybase.org	(Drosophila 12 Genomes Consortium et al. 2007)
Drosophila yakuba	Fruit Fly	1.04	dyak-all-r1.04.gff	flybase.org	(Drosophila 12 Genomes Consortium et al. 2007)
Drosophila ananassae	Fruit Fly	1.04	dana-all-r1.04.gff	flybase.org	(Drosophila 12 Genomes Consortium et al. 2007)
Drosophila persimilis	Fruit Fly	1.3	dper-all-r1.3.gff	flybase.org	(Drosophila 12 Genomes Consortium et al. 2007)
Drosophila pseudoobscura	Fruit Fly	3.03	dpse-all-r3.03.gff	flybase.org	(Richards et al. 2005)
Drosophila willistoni	Fruit Fly	1.04	dwil-all-r1.04.gff	flybase.org	(Drosophila 12 Genomes Consortium et al. 2007)
Drosophila grimshawi	Fruit Fly	1.3	dgri-all-r1.3.gff	flybase.org	(Drosophila 12 Genomes Consortium et al. 2007)
Drosophila mojavensis	Fruit Fly	1.04	dmoj-all-r1.04.gff	flybase.org	(Drosophila 12 Genomes Consortium et al. 2007)
Drosophila virilis	Fruit Fly	1.03	dvir-all-r1.03.gff	flybase.org	(Drosophila 12 Genomes Consortium et al. 2007)
Anopheles gambiae	Malaria Mosquito	4.2	Anopheles-gambiae-PEST_BASEFEATURES_AgamP4.2.gff3	vectorbase.org	(Holt et al. 2002)
Anopheles coluzzii	Mosquito	1.2	Anopheles-coluzzii-Mali-NIH_BASEFEATURES_AcolM1.2.gff3	vectorbase.org	(Lawniczak et al. 2010)
Anopheles merus	Mosquito	2.1	Anopheles-merus-MAF_BASEFEATURES_AmerM2.1.gff3	vectorbase.org	(Neafsey et al. 2015)
Anopheles melas	Mosquito	2.1	Anopheles-melas-CM1001059_BASEFEATURES_AmelC2.1.gff3	vectorbase.org	(Neafsey et al. 2015)
Anopheles quadriannulatus	Mosquito	1.3	Anopheles-quadriannulatus-SANGQUA_BASEFEATURES_AquaS1.3.gff3	vectorbase.org	(Neafsey et al. 2015)
Anopheles arabiensis	Mosquito	1.3	Anopheles-arabiensis-Dongola_BASEFEATURES_AaraD1.3.gff3	vectorbase.org	(Neafsey et al. 2015)
Anopheles christyi	Mosquito	1.3	Anopheles-christyi-ACHKN1017_BASEFEATURES_AchrA1.3.gff3	vectorbase.org	(Neafsey et al. 2015)
Anopheles epiroticus	Mosquito	1.3	Anopheles-epiroticus-Epiroticus2_BASEFEATURES_AepiE1.3.gff3	vectorbase.org	(Neafsey et al. 2015)
Anopheles stephensi	Mosquito	2.2	Anopheles-stephensi-Indian_BASEFEATURES_AsteI2.2.gff3	vectorbase.org	(Neafsey et al. 2015)
Anopheles maculatus	Mosquito	1.3	Anopheles-maculatus-maculatus3_BASEFEATURES_AmacM1.3.gff3	vectorbase.org	(Neafsey et al. 2015)
Anopheles culicifacies	Mosquito	1.3	Anopheles-culicifacies-A-37_BASEFEATURES_AculA1.3.gff3	vectorbase.org	(Neafsey et al. 2015)
Anopheles minimus	Mosquito	1.3	Anopheles-minimus-MINIMUS1_BASEFEATURES_AminM1.3.gff3	vectorbase.org	(Neafsey et al. 2015)
Anopheles funestus	Mosquito	1.3	Anopheles-funestus-FUMOZ_BASEFEATURES_AfunF1.3.gff3	vectorbase.org	(Neafsey et al. 2015)
Anopheles dirus	Mosquito	1.3	Anopheles-dirus-WRAIR2_BASEFEATURES_AdirW1.3.gff3	vectorbase.org	(Neafsey et al. 2015)
Anopheles farauti	Mosquito	2.1	Anopheles-farauti-FAR1_BASEFEATURES_AfarF2.1.gff3	vectorbase.org	(Neafsey et al. 2015)
Anopheles atroparvus	Mosquito	1.3	Anopheles-atroparvus-EBRO_BASEFEATURES_AatrE1.3.gff3	vectorbase.org	(Neafsey et al. 2015)
Anopheles sinensis	Mosquito	2.1	Anopheles-sinensis-SINENSIS_BASEFEATURES_AsinS2.1.gff3	vectorbase.org	(Neafsey et al. 2015)
Anopheles albimanus	Mosquito	1.3	Anopheles-albimanus-STECLA_BASEFEATURES_AalbS1.3.gff3	vectorbase.org	(Neafsey et al. 2015)
Anopheles darlingi	Mosquito	3.3	Anopheles-darlingi-Coari_BASEFEATURES_AdarC3.3.gff3	vectorbase.org	(Marinotti et al. 2013)
Culex quinquefasciatus	West Nile Mosquito	2.2	Culex-quinquefasciatus-Johannesburg_BASEFEATURES_CpipJ2.2.gff3	vectorbase.org	(Arensburger et al. 2010)
Bombyx mori	Silkworm	1.29	Bombyx_mori.GCA_000151625.1.29.gff3	metazoa.ensembl.org	(Xia et al. 2004)
Tribolium castaneum	Red Flour Beetle	3.29	Tribolium_castaneum.Tcas3.29.gff3	metazoa.ensembl.org	(Tribolium Genome Sequencing Consortium et al. 2008)
Nasonia vitripennis	Jewel Wasp	2.29	Nasonia_vitripennis.GCA_000002325.2.29.gff3	metazoa.ensembl.org	(Werren et al. 2010)
Apis mellifera	Honey Bee	4.5	Apis_mellifera.GCA_000002195.1.29.gff3	metazoa.ensembl.org	(Honeybee Genome Sequencing Consortium 2006)
Pogonomyrmex barbatus	Red Harvester Ant	1.2	pbar.genome.OGS.1.2.gff	hymenopteragenome.org	(Smith et al. 2011)
Acyrthosiphon pisum	Pea Aphid	2.29	Acyrthosiphon_pisum.GCA_000142985.2.29.gff3	metazoa.ensembl.org	(International Aphid Genomics Consortium 2010)
Pediculus humanus	Head Louse	2.1	Pediculus-humanus-USDA_BASEFEATURES_PhumU2.1.gff3	vectorbase.org	(Kirkness et al. 2010)
Daphnia pulex	Water Flea (crustacea)	1.29	Daphnia_pulex.GCA_000187875.1.29.gff3	metazoa.ensembl.org	(Colbourne et al. 2011)
Strigamia maritima	Centipede	1.29	Strigamia_maritima.Smar1.29.gff3	metazoa.ensembl.org	(Chipman et al. 2014)

Ixodes scapularis	Deer Tick (arachnid)	1.4	ixodes-scapularis-wikelbasefeaturesiscaw14.gff3	vectorbase.org	(Gulia-Nuss et al. 2016)
Caenorhabditis elegans	Roundworm (nematode)	249	c_elegans.PRJNA13758.WS249.annotations.gff3	wormbase.org	(C. elegans Sequencing Consortium 1998)
Xenopus tropicalis	Western Clawed Frog	4.1	xenTro2.JGI4.1.Aug2005.Ensembl.gtf.gff	genome.ucsc.edu	(Hellsten et al. 2010)
Mus musculus	Mouse	M5	gencode.vM5.annotation.gtf.coding	gencodegenes.org	(Mudge and Harrow 2015)
Tarsius syrichta	Tarsier	1.61	Tarsius_syrichta.tarSyr1.61.gtf.gff	ensembl.org	(Lindblad-Toh et al. 2011)
Homo sapiens	Human	23	gencode.v23.annotation.gtf.coding	gencodegenes.org	(Harrow et al. 2012)
Branchiostoma floridae	Florida Lancelet	1Mar2006	braFlo1Mar2006.OtherRefSeq.gtf.gff	genome.ucsc.edu	(Putnam et al. 2008)
Nematostella vectensis	Sea Anemone	1.29	Nematostella_vectensis.GCA_000209225.1.29.gff3	metazoa.ensembl.org	(Putnam et al. 2007)
Saccharomyces cerevisiae	Baker's Yeast	2June2008	sacCer2June2008Ensembl.gtf.gff	ensemble.org	(Cherry et al. 1997)
Candida albicans	Thrush Yeast	21	C_albicans_SC5314_A21_features_with_chromosome_sequences.gff	candidagenome.org	(Muzzey et al. 2013)
Glycine max	Soybean	1	Glyma1_highConfidence.gff3	phytozome.net	(Schmutz et al. 2010)

Adams MD, Celniker SE, Holt RA, Evans CA, Gocayne JD, Amanatides PG, Scherer SE, Li PW, Hoskins RA, Galle RF, et al. 2000. The genome sequence of Drosophila melanogaster. Science 287:2185–2195.

Arensburger P, Megy K, Waterhouse RM, Abrudan J, Amedeo P, Antelo B, Bartholomay L, Bidwell S, Caler E, Camara F, et al. 2010. Sequencing of Culex quinquefasciatus establishes a platform for mosquito comparative genomics. Science 330:86–88.

C. elegans Sequencing Consortium. 1998. Genome sequence of the nematode C. elegans: a platform for investigating biology. Science 282:2012–2018.

Cherry JM, Ball C, Weng S, Juvik G, Schmidt R, Adler C, Dunn B, Dwight S, Riles L, Mortimer RK, et al. 1997. Genetic and physical maps of Saccharomyces cerevisiae. Nature 387:67–73.

Chipman AD, Ferrier DEK, Brena C, Qu J, Hughes DST, Schröder R, Torres-Oliva M, Znassi N, Jiang H, Almeida FC, et al. 2014. The first myriapod genome sequence reveals conservative arthropod gene content and genome organisation in the centipede Strigamia maritima. PLoS Biol. 12:e1002005.

Colbourne JK, Pfrender ME, Gilbert D, Thomas WK, Tucker A, Oakley TH, Tokishita S, Aerts A, Arnold GJ, Basu MK, et al. 2011. The ecoresponsive genome of Daphnia pulex. Science 331:555–561.

Drosophila 12 Genomes Consortium, Clark AG, Eisen MB, Smith DR, Bergman CM, Oliver B, Markow TA, Kaufman TC, Kellis M, Gelbart W, et al. 2007. Evolution of genes and genomes on the Drosophila phylogeny. Nature 450:203–218.

Gulia-Nuss M, Nuss AB, Meyer JM, Sonenshine DE, Roe RM, Waterhouse RM, Sattelle DB, de la Fuente J, Ribeiro JM, Megy K, et al. 2016. Genomic insights into the Ixodes scapularis tick vector of Lyme disease. Nat. Commun. 7:10507.

Harrow J, Frankish A, Gonzalez JM, Tapanari E, Diekhans M, Kokocinski F, Aken BL, Barrell D, Zadissa A, Searle S, et al. 2012. GENCODE: the reference human genome annotation for The ENCODE Project. Genome Res. 22:1760–1774.

Hellsten U, Harland RM, Gilchrist MJ, Hendrix D, Jurka J, Kapitonov V, Ovcharenko I, Putnam NH, Shu S, Taher L, et al. 2010. The genome of the Western clawed frog Xenopus tropicalis. Science 328:633–636.

Holt RA, Subramanian GM, Halpern A, Sutton GG, Charlab R, Nusskern DR, Wincker P, Clark AG, Ribeiro JMC, Wides R, et al. 2002. The genome sequence of the malaria mosquito Anopheles gambiae. Science 298:129–149.

Honeybee Genome Sequencing Consortium. 2006. Insights into social insects from the genome of the honeybee Apis mellifera. Nature 443:931–949.

International Aphid Genomics Consortium. 2010. Genome sequence of the pea aphid Acyrthosiphon pisum. PLoS Biol. 8:e1000313.

Kirkness EF, Haas BJ, Sun W, Braig HR, Perotti MA, Clark JM, Lee SH, Robertson HM, Kennedy RC, Elhaik E, et al. 2010. Genome sequences of the human body louse and its primary endosymbiont provide insights into the permanent parasitic lifestyle. Proc. Natl. Acad. Sci. U. S. A. 107:12168–12173.

Lawniczak MKN, Emrich SJ, Holloway AK, Regier AP, Olson M, White B, Redmond S, Fulton L, Appelbaum E, Godfrey J, et al. 2010. Widespread divergence between incipient Anopheles gambiae species revealed by whole genome sequences. Science 330:512–514.

Lindblad-Toh K, Garber M, Zuk O, Lin MF, Parker BJ, Washietl S, Kheradpour P, Ernst J, Jordan G, Mauceli E, et al. 2011. A high-resolution map of human evolutionary constraint using 29 mammals. Nature 478:476–482.

Marinotti O, Cerqueira GC, de Almeida LGP, Ferro MIT, Loreto EL da S, Zaha A, Teixeira SMR, Wespiser AR, Almeida E Silva A, Schlindwein AD, et al. 2013. The genome of Anopheles darlingi, the main neotropical malaria vector. Nucleic Acids Res. 41:7387–7400.

Mudge JM, Harrow J. 2015. Creating reference gene annotation for the mouse C57BL6/J genome assembly. Mamm. Genome 26:366–378.

Muzzey D, Schwartz K, Weissman JS, Sherlock G. 2013. Assembly of a phased diploid Candida albicans genome facilitates allele-specific measurements and provides a simple model for repeat and indel structure. Genome Biol. 14:R97.

Neafsey DE, Waterhouse RM, Abai MR, Aganezov SS, Alekseyev MA, Allen JE, Amon J, Arcà B, Arensburger P, Artemov G, et al. 2015. Highly evolvable malaria vectors: The genomes of 16 Anopheles mosquitoes. Science [Internet] 347. Available from: http://www.sciencemag.org/content/347/6217/1258522.abstract

Putnam NH, Butts T, Ferrier DEK, Furlong RF, Hellsten U, Kawashima T, Robinson-Rechavi M, Shoguchi E, Terry A, Yu J-K, et al. 2008. The amphioxus genome and the evolution of the chordate karyotype. Nature 453:1064–1071.

Putnam NH, Srivastava M, Hellsten U, Dirks B, Chapman J, Salamov A, Terry A, Shapiro H, Lindquist E, Kapitonov VV, et al. 2007. Sea anemone genome reveals ancestral eumetazoan gene repertoire and genomic organization. Science 317:86–94.

Richards S, Liu Y, Bettencourt BR, Hradecky P, Letovsky S, Nielsen R, Thornton K, Hubisz MJ, Chen R, Meisel RP, et al. 2005. Comparative genome sequencing of Drosophila pseudoobscura: chromosomal, gene, and cis-element evolution. Genome Res. 15:1–18.

Schmutz J, Cannon SB, Schlueter J, Ma J, Mitros T, Nelson W, Hyten DL, Song Q, Thelen JJ, Cheng J, et al. 2010. Genome sequence of the palaeopolyploid soybean. Nature 463:178–183.

Smith CR, Smith CD, Robertson HM, Helmkampf M, Zimin A, Yandell M, Holt C, Hu H, Abouheif E, Benton R, et al. 2011. Draft genome of the red harvester ant Pogonomyrmex barbatus. Proc. Natl. Acad. Sci. U. S. A. 108:5667–5672.

Tribolium Genome Sequencing Consortium, Richards S, Gibbs RA, Weinstock GM, Brown SJ, Denell R, Beeman RW, Gibbs R, Beeman RW, Brown SJ, et al. 2008. The genome of the model beetle and pest Tribolium castaneum. Nature 452:949–955.

Werren JH, Richards S, Desjardins CA, Niehuis O, Gadau J, Colbourne JK, Nasonia Genome Working Group, Werren JH, Richards S, Desjardins CA, et al. 2010. Functional and evolutionary insights from the genomes of three parasitoid Nasonia species. Science 327:343–348.

Xia Q, Zhou Z, Lu C, Cheng D, Dai F, Li B, Zhao P, Zha X, Cheng T, Chai C, et al. 2004. A draft sequence for the genome of the domesticated silkworm (Bombyx mori). Science 306:1937–1940.
